# The influence of epidural anesthesia on new-born hearing screening: A pilot study

**DOI:** 10.4103/0975-7406.76493

**Published:** 2011

**Authors:** Katijah Khoza-Shangase, Karin Joubert

**Affiliations:** Department of Speech Pathology and Audiology, School of Human and Community Development, University of the Witwatersrand, Johannesburg, South Africa

**Keywords:** Cesarean, epidural, universal newborn hearing screening

## Abstract

**Objective:**

The main aim was to establish if epidural anesthesia had an influence on new-born hearing screening results in newborns born via elective Cesarean section in healthy pregnancies. Specific objectives included determining screening results in a group of newborns born to mothers who had undergone epidural anesthesia during Cesarean section childbirth (experimental group); and comparing the findings with those of a group of newborns born to mothers who had undergone natural delivery without epidural anesthesia (comparison group); while establishing if the time of screening following delivery had any effect on the overall screening results.

**Materials and Methods:**

The above objectives were achieved through the use of a prospective quasi-experimental repeated measures design with a comparison group, where 40 newborns (20 in the experimental and 20 in the comparison group) were screened at three different times through transient otoacoustic emissions (TEOAEs) and automated auditory brainstem response (AABR) measures. All participants were screened while resting quietly in open bassinets in an empty new-born nursery. For both test measures, the results were recorded as either *pass* or *refer*. Data were analyzed through both descriptive and inferential statistics.

**Results:**

Findings indicated that hearing screening earlier than four hours after birth, for both the experimental and comparison groups yielded more false positive findings than testing conducted after 24 hours. An index of suspicion in relation to the influence of epidural anesthesia on Automated Auditory Brainstem Response (AABR), when conducted less than four hours after birth, was raised, as statistically significant findings (*P*<0.05) were obtained.

**Conclusions:**

The findings have implications for timing of screening where universal newborn hearing screening is being implemented.

The slow but unremitting growth of pediatric audiology over the last five decades has culminated in the actuality of delivering services to the youngest and most vulnerable population. This makes preventive audiology a practicable and fundamental foray in current times. Early detection and intervention for hearing impaired infants has become a progressively imperative aspect of neonatal and infant care and has magnified the audiological scope of practice significantly, as a form of secondary prevention.[1] This change in scope of practice has produced a host of new challenges in the delivery of effective and reliable hearing care services to newborns and young infants. This has also resulted in large scale research initiatives that address the rising surge of questions regarding the improvement of methodologies for identification and intervention of hearing loss, most especially in developing countries.[2,3] One of the questions that the current study aims to explore is whether epidural anesthesia has any effect on the hearing screening results in newborns.

The process of identifying the section of population at the highest risk of hearing loss is an essential component of audiological practice and serves as the initial step toward delivering effective audiological services to the pediatric population.[4] The screening of children and infants for hearing loss is a steadily advancing process that has accelerated significantly over the past few years. Even though a number of different methods of detecting hearing loss were evaluated earlier, it was only during the 1990s that substantial progress was made in lowering the average age at which significant hearing loss was identified.[5] This delayed identification of hearing loss was predominantly due to a lack of methodical screening programs and the shortcomings of subjective behavioral screening methods. Fortunately, the emergence of a more accurate, noninvasive, and rapid means of screening hearing loss as well as more concerted and rigorous efforts by professional bodies, such as the Health Professions Council of South Africa,[6] has resulted in the prospect of screening prior to hospital discharge being urged and supported.

As hearing loss cannot be promptly and effortlessly identifiable by routine clinical examinations such as behavioral observation,[7] screening with more objective electrophysiological measures such as otoacoustic emissions and auditory brainstem responses is advocated for the universal screening of all newborns and infants.[5,6] Universal new-born hearing screening programs are aimed at obtaining hearing screening results for every new-born at any time prior to discharge from the hospital.[8] This is believed to possibly be the major way forward to guarantee that early identification and early access to services, including amplification and individualized family-centered early communication intervention, ensues.[9] Early hearing detection through universal newborn screening has taken on exceptional reputation as being the best practice in child healthcare in the developed world;[10] and this highlights the value of ensuring that the methodologies employed are effective and accurate; and yield minimal false positive results in a time frame that is as early as possible.Therefore, it is vital to isolate and categorize factors that may influence the success or failure of new-born audiological screening programs, such as epidural anesthesia, in neonates born to mothers who undergo elective Cesarean section.

False positive results are obtained when a condition is not present, but the test results indicate that it is present. For example, Owen *et al*,[11] document the high number of false positive results when OAEs are obtained in the first 24 hours after birth. Referral rates for OAEs are reported to be 5 – 20% when conducted earlier than 24 hours after birth and less than 3% when conducted 24 to 48 hours after birth.[12] Differing results exist regarding the specificity of OAEs measured at various time intervals after birth. Korres,, *et al*,[13] report a high rate of screening OAE recordings after 24 hours. Research conducted by Levi *et al*,[14] indicates that OAEs can be measured reliably earlier than 48 hours after birth, while Pennsylvania Health Care Cost Containment Counsel[15] suggests that OAEs recorded after 48 hours are more reliable.

Unfortunately, an early discharge from maternity wards may contribute to a significant increase in false positive results due to vernix in the ear canal.[16] Such reports underscore the need for establishing other conditions present early during the postnatal period; such as epidural anesthesia, which may increase the rate of false positive hearing screening findings. Stuart and Moretz[17] suppose that the presence of false positive results may be an important factor hindering the implementation of new-born hearing screening programs. False positive results are also assumed to incur a significant cost to screening procedures;[16] hence, the significance of the current study. According to Gorga *et al*.,[18] an efficient new-born audiological hearing screening program aims to identify newborns with a hearing loss in a cost-effective manner, and incorporates tests that have a low false positive rate. Mehl and Thompson[19] assert that the expense of hearing screening programs also need to be deliberated in terms of the cost of special education and support programs. A delay in the diagnosis of a hearing loss leads to a cost to the general public, the child’s family, and the child with the hearing loss. This is thought to be particularly important considering the documented numbers of hearing impaired individuals.

There is an increasing prevalence rate of hearing loss reported globally.[20] This increasing prevalence has resulted in the customary practice of universal new-born hearing screening in developed countries. Despite South Africa having a comparatively well-developed infrastructure compared to other regions in the Sub-Saharan Africa, new-born hearing screening programs are a long way from common practice. This may be due to the availability of very little contextual research on infant hearing screening. In addition, this lack of data and the increasing priority toward addressing the overwhelming burden of infectious diseases such as HIV / AIDS has raised obstacles in cultivating support, funding, and political activism for infant hearing screening.[20] Therefore, additional research on Early Hearing Detection and Intervention (EHDI) in a South African context is vital for the collation and development of appropriate and efficient neonatal hearing screening guidelines and protocols; hence, the current study.

With the documented prevalence of one to four in every 1000 live births globally, hearing impairment is one of the most common congenital abnormalities in new-borns.[21] Globally, it is reported to be twice as prevalent as other neonatal conditions, screened for at birth.[22,23] The current literature suggests that globally, approximately six in every 1000 infants present with permanent hearing loss at birth or within the neonatal period.[24]

This documented rise in the prevalence rate of hearing impairment worldwide correlates with that reported by the World Health Organization (WHO), which maintains that the estimate for incapacitating hearing loss has increased from 120 million to approximately 278 million in the decade between 1995 and 2005.[25] This increased prevalence is more in developing countries where it is reported that more than 90% of all infants with congenital or early-onset hearing loss reside.[24] In these developing countries, Olusanya and Newton[24] assert that environmental risks are more prevalent, and that early identification programs are exceptionally scarce. Moreover, this reported increased prevalence rate is reported to be even higher, if mild and unilateral hearing losses are also incorporated.[9]

Literature has identified universal new-born hearing screening as the recommended protocol for Early Hearing Detection and Intervention (EHDI), particularly in developed countries.[26] Factors influencing the implementation of universal new-born hearing screening in South African tertiary hospitals include: practicality, ergonomics and economics (cost effectiveness), and the availability of equipment and manpower.[26–28]

If a South African prevalence estimate of 10% is used, an estimated 4.5 million individuals are present with sensorineural hearing loss.[28] This reportedly results in each audiologist being required to serve a significantly larger number of patients when compared to their counterparts in developed countries. Most importantly, the majority of these audiologists operate within the private healthcare sector, where only a small minority of individuals who can afford these services can be seen.[28] Therefore, when matching population size with the number of qualified audiologists in South Africa, there is an apparent scarcity of manpower in the public healthcare sector.[28] This situation is far from being remedied, as formal training in the profession of audiology is also lacking in most tertiary institutions in developing countries.[27]

In comparison to first world countries, the aforementioned factors may influence the ability of audiologists in South Africa to effectively implement universal new-born hearing screening on all infants, prior to hospital discharge. Therefore, the use of targeted screening in high-risk neonates, as well as a clear understanding of the influence that certain postnatal factors (such as epidural anesthesia) may have on the implementation of early hearing screening measures may be more cogent. A need exists to determine the audiological findings in neonates born to mothers who have undergone epidural anesthesia during elective Cesarean sections. It is possible that anesthesia may depress the functioning of the auditory system, as also the integrity of the hearing screening, thereby causing false positive results.[29]

Epidural anesthesia is a procedure that entails the injection of a substance outside the dura mater of the spinal cord, and this causes an autonomic and partial central nervous system blockade. It is commonly used for elective Cesarean section.[30] It has the advantage of allowing the mother to remain awake, minimizes the risk of maternal aspiration, and reduces drug effects on the new-born.[31] The long-term effects of epidural anesthesia, which are rare and minimal, have been well-documented;[30] however, if or how the hearing abilities of new-born infants are affected by epidural anesthesia is not well established.

Some evidence of the influence of anesthesia on new-born infant’s hearing was reported as early as 1988 by Diaz *et al*,[32] examined the effects of maternal lidocaine hydrochlorideanesthesia on the brainstem auditory evoked responses (BAERs)in neonates born by Cesarean delivery. In their study, the findings indicated the effect that maternal anesthesia had on the auditory brainstem response, with a significant delay noted in the central neuralcomponent of the BAER at 90 dB for the experimental group when compared to the control group. In another study by Bozynski *et al*,^33^ the mean wave I-V intervals were prolonged when testing was conducted at less than four hours when compared to findings at 48 hours or longer; and these researchers concluded that changes in the serial auditory brainstem-evoked response tests occurred after maternal lignocaine epidural anesthesia in newborn infants, and that these changes correlated with the blood lignocaine concentrations.

As many studies conducted in the United Kingdom, Israel, and a majority of the developed countries have already established the feasibility of hospital-based hearing screening programs despite early postnatal hospital discharge;[14,34] studies investigating improved methodological strategies need to be prioritized before the realization of effective and efficient low-cost universal new-born hearing screening programs. This includes the appropriate timing of such programs in the postnatal period, to minimize false positive results, while ensuring early identification of neonatal hearing loss prior to hospital discharge.

## Materials and Methods

### Main aim

To establish if epidural anesthesia has an influence on new-born hearing screening results in newborns born via elective Cesarean section in healthy pregnancies.

### Specific objectives

- To determine hearing screening results in a group of newborns born to mothers who had undergone epidural anesthesia during Cesarean section childbirth (experimental group);
- To compare hearing screening findings of the experimental group with those of newborns born to mothers who had undergone natural vaginal delivery without epidural anesthesia (comparison group);
- To establish if the time of the hearing screening following delivery has any effect on the screening results.

### Research design

This study employed a prospective quasi-experimental repeated measures design with a comparison group.[35,36] This design gave the researchers an opportunity to compare the findings of the experimental group with those of the comparison group at different screening times.

### Participants

#### Sample size

The sample comprised of 40 newborns that were born at the chosen private hospital in Johannesburg. The newborns were divided into 20 born to mothers who had chosen to undergo epidural anesthesia during Cesarean section childbirth (experimental group) and 20 born to mothers who had undergone a natural delivery without the use of epidural anesthesia (comparison group).

#### Sampling strategy

A nonprobability sampling strategy, purposive sampling strategy was adopted. The participants were selected from a location convenient to the researchers, where the reported numbers of elective Cesarean section births were high. Participants meeting the inclusion criteria were identified through the Obstetrics and Gynecology Department at the research site and approached to participate in the study.

#### Inclusion criteria

Inclusion criteria for participation were: (i) mothers who had carried the newborns to their full gestational term; (ii) healthy pregnancy; (iii) maternal age less than 35; (iv) no identified risk factors, such as, a number of natural abortions, illness or condition/s that required admission to the Neonatal Intensive Care Unit, craniofacial anomalies, *in-utero* infections, family history of hearing loss; (v) Cesarean section with epidural anesthesia or normal vaginal delivery; and (vi) no epidural anesthesia during natural vaginal delivery.

#### Ethical considerations

Prior to the study being conducted, permission to conduct the study was secured from the Medical Human Research Ethics Committee — Protocol number M070307. Following ethical clearance, the researchers presented the research proposal to the Research Coordinator at the research site for approval to conduct the study. The Review Board at the research site reviewed and approved the study. Thereafter, the participants were invited to volunteer to participate in the study with the assistance of the nursing staff in the maternity ward. The following ethical practices were adhered to during this study:

- Permission to conduct the research was obtained from the gynecological specialist and pediatrician.
- Informed consent was obtained in writing from the mothers of the participants before hearing screening.
- The participants’ rights and worth were respected.
- The voluntary nature of participation was made clear to the participants, and they were notified of their right to withdraw from the study at any point without any negative consequences.
- Confidentiality was ensured through the anonymity of participants and safe storage of information during and after the completion of the research. Research codes instead of participant identifying information were used.
- The researchers were sensitive toward maternal anxiety and when required, in-house counseling services were approached to assist.
- Participants who required further diagnostic testing were provided with both private and public health sector referral details, to ensure that the newborns received early intervention.

### Data collection

#### Materials and procedures

All mothers in the study completed a questionnaire pertaining to the birth time, pregnancy, and birth and family medical history. Information obtained from this questionnaire and from the medical chart reviews allowed the researchers to ensure that the participants met the inclusion criteria and were without risk indicators for hearing loss as defined by the Health Professions Council of South Africa (HPCSA).[6]

Screening measures that were recommended for newborn hearing screening were required to be physiological or objective in nature. These included transient evoked otoacoustic emissions (TEOAE), distortion product otoacoustic emissions (DPOAE), and automated auditory brainstem response (AABR).[27] TEOAEs were low intensity sounds originating from active amplification of the outer hair cells of the cochlear, whereas, DPOAEs were generated by two continuous pure tones presented simultaneously to the ear.[27,37] In contrast, the AABR was an electrical response to auditory stimuli and assessed the integrity and function of the eighth cranial nerve and auditory pathway. OAE and AABR testing modalities were considered to be complementary in nature; hence their combined use in the current study.[38]

All participating newborns were screened using automated TEOAEs (through the use of GSI AUDIO screener) followed by automated ABR (through the use of Maico MB11- MAICO Diagnostic) measures, while resting quietly in open bassinets in an empty new-born nursery. Although both these measures did not require testing to be conducted in a soundproof environment, as they possessed advanced digital signal processing that reduced the effect of ambient noise; the ambient noise levels were monitored through the use of a sound level meter during data collection, to ensure that the findings were valid and reliable and were not negatively influenced by noise. Nursing and sanitary staff were informed about the importance of a quiet environment to obtain accurate results.[18] Every effort was made to minimize physiological noise, by screening newborns when they were resting or immediately after feeding.[39] For both test measures, the results were recorded as either *pass* or *refer* for each participant at each testing session.

#### Data analysis

*Pass / refer* criteria for the analysis of TEOAE and AABR results was adopted. Due to the reported high ambient noise levels in a hospital,[37,40] which primarily affected low frequencies, 250 Hertz (Hz) and 500 Hz were not included within the *pass* / *refer* criteria. Gorga *et al*,^41^ reported noise levels to affect 1 kHz as well.

Following consultation with a statistician, data were analyzed through both descriptive and inferential statistics, utilizing the statistical computer program SPSS. Inferential quantitative analyses of the audiological results were performed using inferential statistical methods, which includedthet-test andFishers Exact test due to their precision in showing relationships in sample sizes below 30.[42] The p-value (0.05) was selected to test the hypothesis with *P*<0.05 indicating rejection of the null hypothesis. The first null hypothesis for the current study was that epidural anesthesia did not have an effect on the hearing screening results of newborns, while its alternate hypothesis was that epidural anesthesia did have an effect. The second null hypothesis was that the time of testing did not have an effect on the results, while its alternate hypothesizes an effect.

#### Reliability and validity

The following measures were adopted to improve the reliability and validity of this study:

Ambient noise levels were minimized and monitored to ensure accuracy of the results[18] and the physiological noise was minimized by only screening the infants when they were resting or immediately after feeding. Furthermore, for TEOAE screening, frequencies below 1 kHz were excluded from the analysis due to these frequencies being most affected by acoustic ambient noise, and external and internal artifacts.The audiological equipment used during the study had undergone the annual calibration prior to data collection, as per the manufacturer’s guidelines, and the biological calibration of the instruments was also performed prior to each testing session. Test administration and control of patient variables was consistent throughout the data collection time, to ensure reliability.[43]

## Results and Discussion

Hearing screening results for the experimental group are depicted in Table 1. These findings indicate that a large majority (90%) of the newborns obtained *refer* results for TEOAEs at sessions 1 and 2 for the experimental group, while 40% obtained the *refer* findings at session 1 for the AABR.

**Table 1:** Hearing screening results in the experimental and comparison group at the two testing sessions

Screening session and measure	Experimental group (n = 20)		Comparison group (n = 20)	
	Pass	Refer	Pass	Refer
Session 1 TEOAEs	10%	90%	20%	80%
Session 1 AABR	60%	40%	90%	10%
Session 2 TEOAEs	10%	90%	20%	80%
Session 2 AABR	100%	0%	100%	0%
Session 3 TEOAEs	100%	0%	100%	0%
Session 3 AABR	100%	0%	100%	0%

AABR: *P* = 0.00001 (< 0.05). The *P* value [*P* = 0.00001 (< 0.05)] rejected the first null hypothesis, thus confirming that epidural anesthesia did have an effect on the AABR hearing screening results of newborns in the current study

The aforementioned results for session 1, although consistent with some previous reports of epidural anesthesia causing delayed latencies on the ABR should be interpreted with caution as the presence of vernix in the neonates could have had an additional influence on the AABR. Nonetheless, the fact that vernix could have also had a similar influence on the AABR in the comparison group, but did not, raises a strong index of suspicion about the role of epidural anesthesia, which was the only differentiating factor between the two groups. Of particular interest is the significant improvement in *pass* AABR screening results at sessions 2 and 3; possibly indicating that the effects of the anesthesia may have worn off by then. The same cannot be said for the OAE screening results, which seemed to remain fairly the same at sessions 1 and 2 for both groups; with clear significant changes at session 3. The TEOAE findings confirm documented evidence that OAE screening is more reliable 24 hours after birth, due to vernix.

### Comparison of hearing screening findings of the experimental group with the comparison group

**Figure 1:**
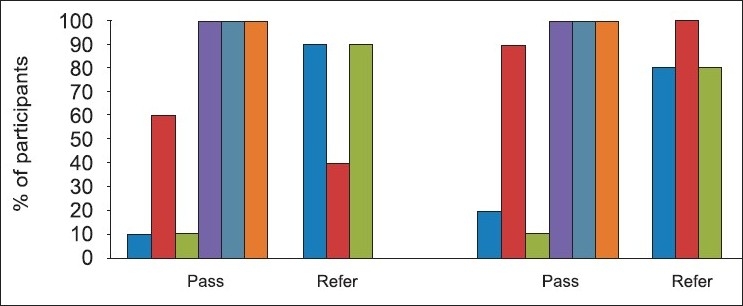
Comparing the experimental and comparison results

Overall, as depicted in Table 1 and Figure 1, a large majority of newborns in both groups obtained *refer* TEOAE findings at the earlier testing sessions. The TEOAE hearing screening findings of both groups only changed when screening was conducted after 24 hours (at discharge). The AABR findings indicated a higher (40% at session 1) *refer* rate in the experimental group when compared to the 10% in the comparison group. The AABR findings positively changed with all newborns who had obtained *refer* results at session 1 passing at sessions 2 and 3.

These findings may indicate that even though the effective use of OAEs and AABR as screening measures has been well established, it is important to establish factors that may influence the reliability of these measures. In the current study, epidural anesthesia seems to have had an influence with regard to increasing false positive findings when testing was conducted earlier than four hours after birth.

### Establishing if the time of hearing screening following delivery has any effect on the screening results

The p-values of 0.00014 for a two-sided exact significance and 0.00007 for a one-sided exact significance were found when examining if time of screening following delivery had any effect on the results. These values reject the null hypothesis (*P*<0.05), hence indicating that time of testing did have an effect on the screening results in the current study.

From the results obtained, it can be concluded that TEOAE testing earlier than four hours after birth, as well as between four and six hours after birth is unfavorable. This was postulated to be possibly due to the vernix present in the external auditory canal at such times. According to Korres *et al*,[13] and the Pennsylvania Health Care Cost Containment Counsel,[15] TEOAEs are viable tools during new-born hearing screening between 24 and 48 hours after birth, because at that time the external auditory canal would be free of vernix. An index of suspicion about the influence of epidural anesthesia was raised.

## Conclusions

Despite the fact that the sample size for the current study was small; and therefore limited the generalizability of the results, findings from the current study have significant implications for the implementation of universal new-born hearing screening programs; particularly in developing countries, where allocation of resources is driven by priorities such as management of infectious conditions such as the HIV / AIDS pandemic. Knowing where and when to focus the available resources for the best and effective EHDI programs would not only improve service delivery; but may improve access by the general South African population to the services of audiologists; which are currently scarce, particularly in the public healthcare sector. From the current findings, evidence points to the reliability of performing hearing screening with AABR on the day of delivery, as long as that happens four hours following the birth, in newborns where anesthesia was used during delivery. The timing of universal hearing screening, especially in babies born with epidural anesthesia, is important, as the use of epidural anesthesia could lead to increased false positive results, which may therefore cause undue maternal anxiety. The use of OAEs seems to be significantly influenced by vernix in the first few hours following birth, highlighting the need for ensuring that fluids in the external auditory canal are actively cleared before reliable use of OAEs can be implemented as a screening measure before 24 hours. Findings from the current study have particular relevance in developing countries such as South Africa, where women attending state hospitals’ maternity wards are discharged with their babies a few hours after giving birth. The current study should be replicated within a larger sample size with diagnostic ABR and OAEs, where findings will not be restricted to *pass* or *refer*; but would provide specific findings about the site and degree of the influence of anesthesia on the auditory pathway.
